# P-2068. Area Deprivation Is Linked to Prevalence of Multidrug Resistance in Enterobacterales Clinical Isolates

**DOI:** 10.1093/ofid/ofaf695.2232

**Published:** 2026-01-11

**Authors:** Heather Henderson, Billy J Williams, Kevin Alby, David van Duin

**Affiliations:** University of North Carolina at Chapel Hill, Chapel Hill, NC; UNC Hospital, Chapel Hill, North Carolina; University of North Carolina, Chapel Hill, North Carolina; University of North Carolina at Chapel Hill, Chapel Hill, NC

## Abstract

**Background:**

Antimicrobial resistance is a global health emergency that disproportionately affects vulnerable populations. We evaluated whether the prevalence of multidrug resistance (MDR) in Enterobacterales clinical isolates varied according to neighborhood deprivation level among patients of a health system in North Carolina.

**Table:**
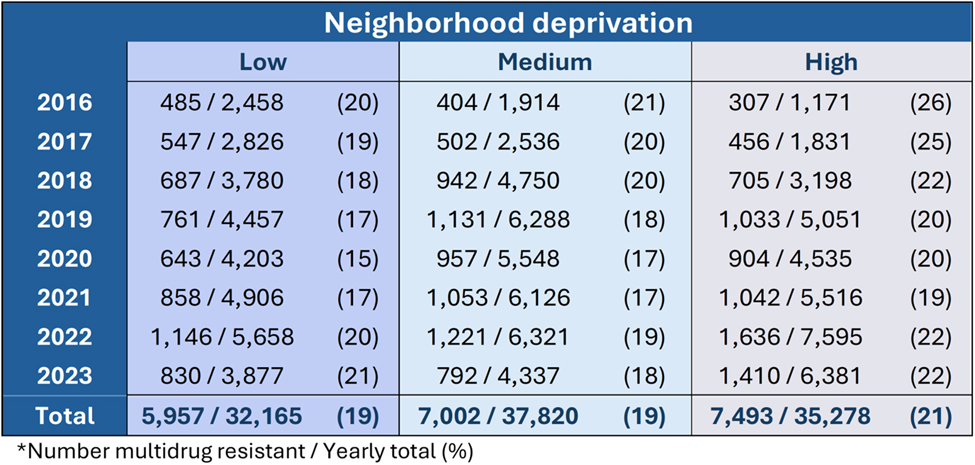
Multidrug resistance* among Enterobacterales isolates, by year and neighborhood deprivation level

**Figure:**
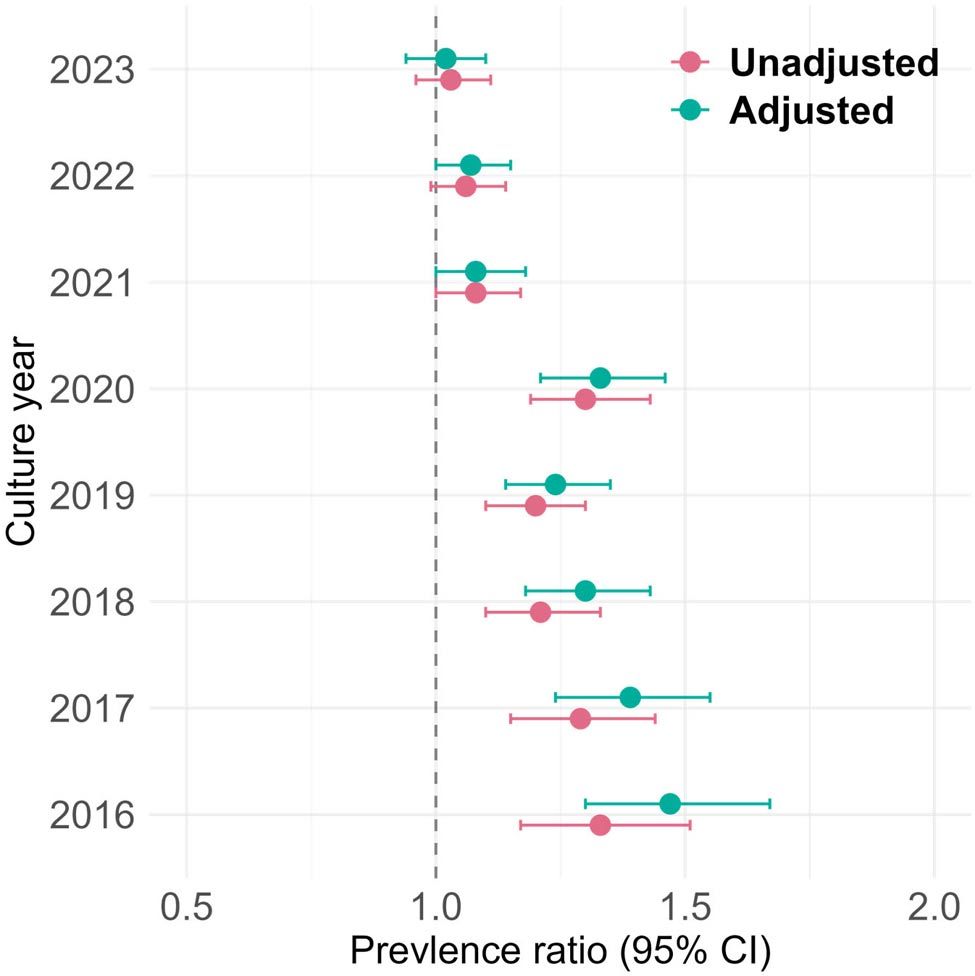
Prevalence ratios for multidrug resistance in high- vs low-deprivation neighborhoods, by year

**Methods:**

The study population included all adult patients with an Enterobacterales clinical isolate from 2016–2023. We analyzed microbiological data from the UNC Health system clinical laboratory and demographic data from the electronic health record. Isolates non-susceptible to ≥ 3 antibiotic classes were defined as MDR. Neighborhood deprivation was assigned based on patient ZIP code of residence using the Area Deprivation Index (ADI), categorized as “low,” “medium,” or “high” deprivation (ADI deciles 1-3, 4-7, and 8-10, respectively). We estimated unadjusted and adjusted prevalence ratios for MDR comparing patients from high- vs low-deprivation neighborhoods. Multivariable models included age, gender, race, ethnicity, specimen source (blood, respiratory, urine, other), and location type (inpatient, outpatient, emergency department, intensive care).

**Results:**

We identified 105,263 unique Enterobacterales isolates from 85,847 patients; 20,452 of all isolates (19%) were MDR. The prevalence of MDR was consistently higher among patients from high- vs low-deprivation neighborhoods across all years, although the differences narrowed over time (Table). A decreasing trend in adjusted prevalence ratios (aPR) was observed (Figure), with aPR = 1.5 (95% CI, 1.3-1.7) in 2016 and aPR = 1.0 (95% CI, 0.9-1.1) in 2023. An exception to the decreasing trend occurred in 2020, with aPR = 1.3 (95% CI, 1.2-1.5). In multivariable models, the prevalence of MDR was significantly increased among patients from high-deprivation neighborhoods for all years except 2023.

**Conclusion:**

Isolates from patients living in more deprived neighborhoods had a higher prevalence of MDR compared with those from less deprived areas. This excess prevalence decreased over time – with a temporary increase coinciding with the onset of the COVID-19 pandemic – and was no longer observed in 2023. Further research is needed to understand the drivers of both the excess prevalence and the recent reduction.

**Disclosures:**

Kevin Alby, PhD, Gradientech: Grant/Research Support David van Duin, MD, PhD, British Society for Antimicrobial Chemotherapy: Editor stipend|Merck: Advisor/Consultant|Merck: Grant/Research Support|Pfizer: Advisor/Consultant|Roche: Advisor/Consultant|Shionogi: Advisor/Consultant

